# Supplementary material for: Targeting triple‐negative breast cancer with an aptamer‐functionalized nanoformulation: a synergistic treatment that combines photodynamic and bioreductive therapies
Source: J Nanobiotechnology. 2021 Mar 29;19:89. doi: 10.1186/s12951-021-00786-8 (PMC8008604; doi:10.1186/s12951-021-00786-8)
Supplement: Supplementary file 1 — Additional file 1: Figure S1 XRD pattern of MMT-2. Figure S2. The cytotoxicity of two mesoporous silica nanoparticles (MMT-2, and LXL-1-MMT-2) on MDA-MB-231 cells. Various concentrations of nanoVector (0, 1.12, 2.24, 4.48, 8.96, and 17.92 g/mL) were used to treat cells. Cell viability was assessed after 24 h of treatment. Each bar represents an average of at least three repetitive analyses. Figure S3. Quantitative data of singlet oxygen molecules generated by photoexcitation of PpIX. The singlet oxygen molecules generated by 630 nm of LED in a DPBF quantitative PpIX. (a) The concentrations of PpIX were 0, 0.2, 0.4 and 0.8 µM, and the irradiation times were 10, 20, and 30 sec. (b) The oxygen concentrations were 0 %, 1 %, 5 %, and 21 %, the PpIX concentration was 0.8 µM, and the irradiation time was 10, 20, and 30 sec. (c) DPBF concentration of 250 µM, PpIX concentration of 0.4 µM, PpIX-MMT2 concentration of 1.17 g/mL, LXL-1-PpIX-MMT-2 concentration of 1.17 g/mL, and irradiation times of 0, 10, 20, 30, 40, and 50 sec. Each data point represented an average of three replicates. Figure S4. Quantification of ROS produced by cells irradiated with PpIX at different oxygen concentrations with different lengths of time of irradiation. The concentration of PpIX was 0.4 µM, DCFDA concentration was 40 µM, and the irradiation times were 0, 1, 2, 3, and 4 min. All data represented an average of three replicates. Figure S5. Photo illustration of LED light sources. (a) LED light device that was suitable for use in 96 microplate format. (b) The irradiation of cells on a 96-well plate was achieved by stacking the LED light device (as seen in a) and black 96-well plate (for guiding light) on the white plate cultured with various groups of cells (c) The LED device that was used in animal studies. [file 12951_2021_786_MOESM1_ESM.pdf]

# Targeting Triple-Negative Breast Cancer with an Aptamer-Functionalized Nanoformulation: A Synergistic Treatment that Combines Photodynamic and Bioreductive Therapies

**Yi-Te Chou,[¶,1] Chih-Yu Lin,[¶,2] Jyun-Wei Wen[¶,1], Ling-Chun Hung[1,3], Ying-Feng Chang[1], Chia-Min Yang,[2,4]*** Li-chen Wu,[5]** Ja-an Annie Ho[1,6,7]***

[1]BioAnalytical Chemistry and Nanobiomedicine Laboratory, Department of Biochemical Science and Technology, National Taiwan University, Taipei, 10617 Taiwan

[2]Department of Chemistry, National Tsing Hua University, Hsinchu, 30013 Taiwan

[3]NTU Instrumentation Center, Technology Commons, College of Life Science, National Taiwan University, Taipei, 10617 Taiwan

[4]Frontier Research Center on Fundamental and Applied Sciences of Matters, National Tsing Hua University, Hsinchu, 30013 Taiwan

[5]Department of Applied Chemistry, National Chi Nan University, Puli, Nantou, 54561 Taiwan

[6]Biotechnology Center, National Taiwan University, Taipei, 10617 Taiwan

[7]Department of Chemistry, National Taiwan University, Taipei, 10617 Taiwan

Manuscript submitted to ***Journal of Nanobiotechnology***

[¶]Authors contributed equally.

*Corresponding authors. E-mail addresses: cmyang@mx.nthu.edu.tw (Chia-Min Yang); lw25@ncnu.edu.tw (Li-chen Wu); jaho@ntu.edu.tw (Ja-an Annie Ho)

## ABSTRACT

**Background:** Areas of hypoxia are often found in triple-negative breast cancer (TNBC), it is thus more difficult to treat than other types of breast cancer, and may require combination therapies. A new strategy that combined bioreductive therapy with photodynamic therapy (PDT) was developed herein to improve the efficacy of cancer treatment. Our design utilized the characteristics of protoporphyrin IX (PpIX) molecules that reacted and consumed $O_2$ at the tumor site, which led to the production of cytotoxic reactive oxygen species (ROS). The low microenvironmental oxygen levels enabled activation of a bioreductive prodrug, tirapazamine (TPZ), to become a toxic radical. The TPZ radical not only eradicated hypoxic tumor cells, but it also promoted therapeutic efficacy of PDT.

**Results:** To achieve the co-delivery of PpIX and TPZ for advanced breast cancer therapy, thin-shell hollow mesoporous *Ia3d* silica nanoparticles, designated as MMT-2, was employed herein. This nanocarrier designed to target the human breast cancer cell MDA-MB-231 was functionalized with PpIX and DNA aptamer (LXL-1), and loaded with TPZ, resulting in the formation of TPZ@LXL-1-PpIX-MMT-2 nanoVector. A series of studies confirmed that our nanoVectors (TPZ@LXL-1-PpIX-MMT-2) facilitated *in vitro* and *in vivo* targeting, and significantly reduced tumor volume in a xenograft mouse model. Histological analysis also revealed that this nanoVector killed tumor cells in hypoxic regions efficiently.

**Conclusion:** Taken together, the synergism and efficacy of this new therapeutic design was confirmed. Therefore, we concluded that this new therapeutic strategy, which exploited a complementary combination of PpIX and TPZ, functioned well in both normoxia and hypoxia, and is a promising medical procedure for effective treatment of TNBC.


**Key words:** Triple-negative breast cancer, photodynamic therapy, tumor hypoxia, bioreductive prodrug, hollow mesoporous silica nanoparticle, DNA aptamer

## Background

Breast cancer was ranked as having the highest cancer mortality for women globally in 2020 [1]. About 10-20% of breast cancers are triple-negative breast cancers (TNBC), which shares several similar characteristics with basal-like subtypes, and is commonly associated with *BRCA1* gene mutations [2-5]. TNBC was named because it lacked the expression of progesterone receptors (PR), estrogen receptors (ER), and human epidermal growth factor receptor type 2 (HER2). Patients with TNBC may experience increased incidence of distant metastasis, early recurrence, and higher mortality than other breast cancer subtypes [3, 4].

TNBC remains a challenging subtype of breast cancer to treat, and its high rates of relapse are likely due to the presence of increased levels of cancer stem cells [6-8]. A lack of well-defined molecular targets in TNBC has resulted in limited therapeutic options. Therefore, identification of new therapeutic targets for TNBC is a high clinical priority. Therapeutics to target oncogenic signaling pathways, which include the PI3K/AKT/mTOR pathway and Src/Wnt signaling [9-11], have been investigated for their effectiveness in the treatment of TNBC. In addition, the dysfunction of BRCA1/2 has also been used as an index to predict treatment response [12]. Moreover, combination drug therapy that used targeted cancer drugs and chemotherapy have been investigated in clinical trials as well [9]. Unfortunately, the treatment outcomes for patients varied significantly because of the heterogeneity observed among breast cancers. Alternative therapeutic strategies with improved efficiency are thus in urgent demand.

Hypoxia-inducible factor 1 (HIF1) is a transcription factor, which consists of an oxygen-sensitive α subunit and a β subunit that allows cellular adaption to hypoxia. An elevated expression of HIF1-α in TNBC was evident, which demonstrated that TNBC often grew under hypoxic conditions [9, 13]. Accordingly, targeting hypoxic cancer cells seems to be a plausible idea for treating TNBC. This phenomenon inspired us to utilize bioreductive drugs (BD) agents, which are inactive prodrugs that can be converted into potent cytotoxins under conditions of either low oxygen tension or in the

presence of high levels of specific reductases, to develop new therapeutic strategies. Rapidly growing tumor cells are often exposed to hypoxia, a common sign of stress. Tumor cells may multiply farther away from the blood supply, which results in a relatively low oxygen tension of <8 mm Hg (1 %) compared with a normal blood oxygen pressure of 70 mm Hg or 9.5 % [14].

Photodynamic therapy (PDT), one of the clinically approved treatments, is a minimally invasive approach for treating various cancers [15]. The mechanism of PDT is based on the activation of photosensitizers (PSs) to the excited singlet state after visible light absorption, followed by intersystem crossing to the excited triplet state [16]. The excited PSs undergo photochemical reactions with oxygen ($O_2$) to form cytotoxic reactive oxygen species (ROS) [17], which not only eradicates cancer cells, but also normal cells such as blood vessel epithelial cells. PDT has been implemented as an anti-breast cancer strategy [18], but the hypoxic tumor microenvironment in tumorous breast tissues often impeded the utility of PSs, such as PpIX, and caused undesirable consequences, such as angiogenesis, low rate of cancer cures, and increased likelihood of tumor recurrence [19]. In other words, PDT alone may enable a blockade of nutrients and oxygen in the cancerous areas, resulting in the killing of cancerous cells, but the formation of hypoxic areas somehow alters cancer cell metabolism and may thereby contribute to therapy resistance [20]. Therefore, it is clear that tumor hypoxia remains as one of the greatest challenges in treating solid tumors because cancer cells in such regions are a potent barrier to effective radiation therapy and immunotherapy [21]. Since oxygen consumption is a limiting factor for PDT [22], several strategies have been developed to improve its therapeutic efficacy to increase radical formation. Xia and co-workers [23] introduced oxygen-independent free radicals produced by a polymerization initiator system to destroy hypoxic cancer cells. Additionally, BD or hypoxia-activated prodrugs (HAP) have also been alternatives to eliminate hypoxic cancer cells.

It is known that both BD and HAP are inactive but can be converted into potent toxins under conditions of either low oxygen tension or in the presence of high levels of specific reductases [24, 25]. For example, the cytotoxicity of drugs RB-6145, SR-4233 (TPZ), and E09 (Aqaziquone) that were used

in a hypoxic environment, was approximately 50–200 fold higher than that in an aerobic environment [26]. TPZ is a class of cytotoxic drugs with selective toxicity towards hypoxic mammalian cells that can be catalyzed by NADPH: cytochrome c (P450) reductase to form toxic hydroxyl and benzotriazinyl radicals, followed by the generation of ROS to damage DNA when a cell was deprived of oxygen [27]. TPZ has been evaluated in clinical trials in non-small cell lung cancer, head and neck cancer, cervical cancer, and metastatic melanoma [25]. Regrettably, it did not show satisfactory results as originally anticipated in clinical trials due to low cellular uptake efficiency, unsatisfied pharmacokinetics, and adverse side effects [25]. Based on the hypoxic tendency of TNBC and the complementary functions of PS and BD, we were motivated to target normoxic and hypoxic tumor areas by adopting PDT in conjunction with bioreductive therapy to evaluate the synergistic antitumor effects of this new nanoVector-assisted therapeutic strategy for TNBC. Co-delivery of PpIX and TPZ can be realized readily using hollow mesoporous silica nanoparticles (HMSNs), which is an ideal type of drug carrier because of its biocompatibility, degradability, high loading capacity and versatile surface chemistry [28-32].

In this study, MMT-2, a novel type of thin-shell HMSNs with three-dimensionally interconnected mesopores we previously developed [33], was applied to integrate the therapeutic utilities of PpIX and TPZ and the targeting capability of the DNA aptamer LXL-1 for TNBC cell line MDA-MB-231. PpIX and LXL-1 were modified covalently on the mesopores and external surface of MMT-2, respectively, and TPZ was finally loaded largely into the hollow interior of the functionalized MMT-2, designated as LXL-1-PpIX-MMT-2, by impregnation (**Scheme 1**). *In vitro* and *in vivo* studies showed that the obtained nanoVector TPZ@LXL-1-PpIX-MMT-2 was accumulated selectively at the tumor site and demonstrated high efficacy in killing MDA-MB-231 cells in normoxic and hypoxic areas under 630 nm irradiation; the synergistic effect was manifested clearly in a complete tumor eradication with enhanced efficiency. Our results confirmed that this reliable nanomedical platform offers a promising strategy for TNBC targeted therapy, and it provides a solution for limited therapeutic efficacy that is often associated with PDT due to the deprivation of oxygen level in cancer cells.

## Results and Discussion

### ***Characterization of materials***

The as-synthesized MMT-2 displayed a characteristic XRD (X-ray diffraction) pattern that corresponded to *Ia3d* symmetry (**cf. Figure S1**), and the hollow morphology and the thin shell with ordered mesostructure of each nanoparticle could be observed by TEM (transmission electron microscopy) (**Figure 1a**). The ordered mesostructure was retained after subsequent steps of functionalization, as evidenced by the TEM image of LXL-1-PpIX-MMT-2 (**Figure 1b**). Successful functionalization of maleimide groups on the external surface and PpIX on the mesopores were confirmed by TGA (thermogravimetric analysis) and FTIR (Fourier-transform infrared spectroscopy), and the final conjugation of the DNA aptamer LXL-1 with maleimide groups was supported by the change in surface potential. The relative organic content of M-PpIX-MMT-2 (maleimide functionalized, PpIX-anchored MMT-2) was higher than that of M-MMT-2 (maleimide functionalized MMT-2), which showed weight losses of 29.8 wt% for M-PpIX-MMT-2 and 12.7 wt% for M-MMT-2, as revealed by TGA (**Figure 1c**). Characteristic IR signals confirmed the presence of the maleimide groups and PpIX (**Figure 1d**). The amount of PpIX in M-PpIX-MMT-2 was estimated to be ~0.343 mole per gram of the sample by analyzing the absorbance at 405 nm [34] in the UV-vis spectrum of the sample. After conjugation of the highly negatively charged DNA aptamer, the zeta potential measured in PBS changed from -19 mV for M-PpIX-MMT-2 to -38 mV for LXL-1-PpIX-MMT-2 (**Figure 1e**). The hydrodynamic size of LXL-1-PpIX-MMT-2 was around 345.0 ± 3.4 nm as measured by dynamic light scattering (DLS) (**Figure 1e)**. In addition, the cell viability of MMT-2 and LXL-1-MMT-2 on MDA-MB-231 cells was shown in **Figure S2**.

### ***Cellular uptake and in vivo targeting efficiency of LXL-1-PpIX-MMT-2***

The cell uptake and targeting efficiency of LXL-1-PpIX-MMT-2 toward MDA-MB-231 breast cancer cells (a TNBC cell line) were investigated. After treating with either free PpIX or LXL-1-PpIX-MMT-2 for 5 h, cells were harvested and lysed to determine the uptake of PpIX by a fluorospectrometer. In addition,

a Laser Confocal Microscope was used to monitor targeting efficiency. (**Figure 2a**). The LXL-1-PpIX-MMT-2-treated group demonstrated four times more PpIX accumulation than that of the cell group treated with PpIX alone. The confocal microscopy imaging also indicated that cells treated with LXL-1-PpIX-MMT-2 (for 5 h) showed greater enhanced fluorescence than that in the free PpIX-treated group (**Figure 2b**). These results suggested that LXL-1-PpIX-MMT-2 was able to be taken up and accumulated in MDA-MB-231 cells (**Figure 2a, b**).

Next, the targeting efficiency of LXL-1-PpIX-MMT-2 was studied. Three breast cancer cells were used, which included (1) MDA-MB-231, (2) MCF-7 that is a human breast cancer cell line with estrogen, androgen, progesterone, and glucocorticoid receptors, and (3) MCF-10A that is a non-tumorigenic epithelial cell line. The highest amount of PpIX accumulated in MDA-MB-231 cells among all tested cell groups (**Figure 2c**). Similar results were seen in confocal microscopy images, where MDA-MB-231 cells revealed the highest fluorescence among tested cells after being treated with LXL-1-PpIX-MMT-2 (**Figure 2d**). These results confirmed the selective targeting ability of LXL-1-PpIX-MMT-2 toward the TNBC cell line MDA-MB-231.

Due to structural characteristics, the hydrophobic PpIX is supposedly to deposit in hepatic rather than renal excretion [35]. Our LXL-1-PpIX-MMT-2 was designed to increase PpIX accumulation in tumor, but to reduce in the other organs. *In vivo* targeting was revealed by an IVIS (**Figure 2e**). Aside from the tumor site, free PpIX accumulated significantly in the liver, lung and kidney. On the other hand, LXL-1-PpIX-MMT-2 was apparently retained in the tumor, and somewhat less in the others. There was no doubt that our LXL-1-PpIX-MMT-2 helped to deliver the cargo drug to the targeted region, which enhanced the accumulation of the PS in the tumor. These results showed that LXL-1-PpIX-MMT-2 was able to target MDA-MB-231 xenografts without significant residuals in other organs, which promoted safety and therapeutic efficacy.

***Effect of oxygen level on photodynamic cytotoxicity***

Considering the basic principles of PDT, factors including oxygen level, irradiation time, and the concentration of photosensitizer are presumably key elements that decide the efficacy of PDT. Optimal conditions to maximize the effectiveness of PDT were, therefore, investigated. **Figure S3** showed the efficiency of PpIX for generating singlet oxygen upon irradiation under different oxygen level. **Figure S4** revealed the effect of oxygen level on *in vitro* production of ROS in cancer cells. In addition, MDA-MB-231 cells were cultured at either a normal oxygen level or under hypoxic conditions (at 5%, 2%, and 1% oxygen) to examine the minimum oxygen level to obtain an acceptable photodynamic therapeutic outcome. Significant photodynamic cytotoxicity of PpIX at oxygen level of 21% and 5% was observed, whereas reduced cytotoxicity was exhibited under hypoxic conditions (2% and 1% oxygen level) (**Figure 3**). With sufficient oxygen supply, cytotoxicity increased with the elevated amount of photosensitizer and irradiation time. In contrast, under hypoxic conditions, decreased cytotoxicity occurred at a relatively high photosensitizer concentration (0.8 μM of PpIX, equivalent to 0.46 μg/mL) and long irradiation period (4 min). Our results agreed with previous studies [36] indicating that satisfactorily high oxygen level was required to photoactivate PpIX to induce photodynamic cytotoxicity. Based on our results, 0.4 μM PpIX at 2% oxygen was able to eradicate ~50% of treated cells, thus, 0.4 μM PpIX was selected for further study (**Figure 3b**).

***Effect of oxygen level on TPZ cytotoxicity***

We know that high levels of oxygen caused cytotoxic TPZ radicals to become less harmful TPZ molecules. The low oxygen level ranging from 0.3% to 4.2% in tumor microenvironments has been discussed extensively [14], which encouraged us to examine TPZ cytotoxicity under various hypoxic conditions (oxygen levels: 1%, 2%, 5%) and under normoxia (oxygen level: ~21%). As anticipated, the observed cytotoxicity was enhanced with the increase in TPZ concentration and lower oxygen level (**Figure 3e**). TPZ displayed low toxicity (cell viability ~80% at 60 μM) at a high oxygen level (~21%). With a limited supply of oxygen, improved cytotoxicity was revealed even at a low TPZ concentration (cell

viability ~50% at 20 µM). In addition, significant cytotoxicity (<50% cell viability) was found at a higher TPZ concentration (60 µM, equivalent to 11 µg/mL) with low oxygen levels (such as, 5%, 2%, and 1%). Therefore, a TPZ concentration of 60 µM was identified as the optimum effective dosage for further studies. Our observations agreed with the results as reported in previous studies [24, 37, 38], in which the cytotoxicity of TPZ was inversely associated with oxygen level.

***Synergistic effect of PDT- and TPZ-based combination therapy***

The antitumor effects of PDT highly depend on the tumor oxygen level, but are hindered by hypoxic tumor microenvironments. To improve poor effectiveness of PDT associated with tumor hypoxia, we established a new therapeutic approach that combined two cancer drugs that work in a complementary fashion. PDT requires sufficient oxygen to generate toxic radicals that are harmful to tumor cells, so bioreductive prodrugs that can be activated to be highly toxic under low-oxygen conditions were a perfect match. Therefore, the combination treatment of PpIX and TPZ was conducted *in vitro*. With the elevated oxygen level, the cytotoxicity of PpIX and TPZ showed opposite trends (**Figure 4a**). Cell viability increased from 31% to 88% for the PDT-only group with the lower oxygen level (5% to 1%), but cell viability in the TPZ-only group decreased from 42% to 35% (oxygen level from 5% to 1%). Once we combined free PDT with free BD, elevated cytotoxicity was observed for all groups, in general, at different oxygen levels. However, cell viability increased from 4% to 22% with the decrease in oxygen level from 5% to 1%, which indicated that PDT played a dominant role in determining therapeutic efficacy. Moreover, the synergistic effect provided by this new combination treatment was observed because CDI (coefficient of drug interaction) values of 0.3, 0.49, and 0.7 were obtained at oxygen levels of 5%, 2%, and 1%, respectively, whereas CDI values that were more than or equal to one indicated antagonistic or additive effects, respectively [39] (**Figure 4b)**. It was also claimed previously [37, 40] that the combination of PDT and HAP prodrugs increased cell cytotoxicity synergistically.

Furthermore, it is noteworthy that the combination treatment with two free drugs exhibited less cytotoxicity at a low oxygen level of 1% compared with higher oxygen levels (5% and 2% $O_2$); however, the combination treatment with our nanoVector, TPZ@LXL-1-PpIX-MMT-2, further decreased cell viability not only at the 1% oxygen level, but also at 2% and 5%, which holds promising potential to be used in hypoxic environments for tumors. We believe that this was due to the effective targeted delivery of PpIX and TPZ to MDA-MB-231 cells.

Although numerous reports have demonstrated evidences on the utilities of nanoDrug Delivery Systems *in vitro* and/or *in vivo*, limited research was conducted to evaluate therapeutic efficacy of nanotherapy on hypoxia formation and cytotoxicity in hypoxic regions. The use of nanoVector-mediated combination therapy based on the complementarity of PDT and BD to enhance therapeutic efficacy against cancer, especially for tumor hypoxia, was addressed herein. We again confirmed that low oxygen level impaired PDT cytotoxicity, but promoted the activity of TPZ (**cf. Figure 3, 4**), which was in agreement with previous findings [25, 38, 40, 41].

TNBC is aggressive with high mortality and difficult to treat [42]. The unsatisfactory therapeutic outcomes of conventional chemotherapy and therapeutic agents, primarily poly(ADP-ribose) polymerase inhibitors and EGFR inhibitors, argue for development of an effective targeted therapy for this ER/PR/HER2 receptor expression-lacking tumor. A genetic mutation in p53 has been revealed recently in TNBC that could be a therapeutic target [43]. Interestingly, the cytotoxicity of TPZ was observed previously in p53-dysfunctional epidermoid carcinoma (A431) cells [41]. In fact, there are a number of studies that utilized TPZ in combination with cisplatin to treat head and neck cancer, lung cancer, and breast cancer [44]. The utility of our nanoVector, together with findings obtained from previous studies [40, 41], validated the effectiveness of PDT/BD combination therapy to eradicate cancer cells with the *TP53* mutation, which offers an alternative approach for TNBC treatment.

***Antitumor activity of LXL-1-PpIX-MMT-2 in a MDA-MB-231 xenograft tumor model***

Conventionally, chemotherapy is often given after surgery because information collected from post-surgical pathology is necessary to determine the optimum regimen for cancer treatment. Today, given the increasing interest in local/regional therapy, localization of the tumor is feasible [45]. Numerous molecular approaches for diagnosis and characterization of breast tumors are available to provide detailed information to predict chemotherapy outcomes before surgery [46]. With the precise localization of tumors, we believe that the direct injection of chemotherapeutic drugs at the site of the tumor will enable the relief of serious systematic toxicity caused by the drugs themselves. Accordingly, intratumoral administration was performed in our *in vivo* study, which attempted to further improve the survival and quality of life for patients.

To evaluate further the therapeutic effectiveness of this novel nanotherapeutic strategy, we used NU/NU female mice (4 wk old) that carried human breast tumor xenografts in two thighs. NanoVectors and free drugs were administrated i.t. as described previously (**Figure 5a**). Treatment with TPZ@LXL-1-PpIX-MMT-2 demonstrated the best therapeutic efficacy among all experimental animal groups (**Figure 5b, c**). Additionally, no significant body weight loss was observed during the study period (**Figure 5d**). Moreover, as evidenced by H&E staining (**Figure 5e**), tumors treated with our nanoVectors showed reduced cell density compared with those groups treated with single free drugs (PpIX or TPZ), or a combination of free drugs (PpIX + TPZ). The tumor hypoxic area was also examined by immunohistochemical staining of pimonidazole–protein adducts in hypoxic areas (**Figure 5e**). The hypoxic zone in the PpIX-treated group was larger than that of the PBS-treated and TPZ-treated groups. TPZ@LXL-1-PpIX-MMT-2 not only restrained the formation of notable hypoxia, but also promoted cell death in the same region as observed by reduced cell density compared with the PBS group. PDT increased hypoxia due to its inherent cytotoxic mechanism, where photosensitizers interacted with oxygen to form ROS that led to the formation of a hypoxic tumor microenvironment.

In summary, MMT-2 comprising thin-shell hollow mesoporous silica nanoparticles was selected as the drug vector for PDT/BD combination therapy. The material featured large hollow interior, thin

mesoporous shell and uniform particle size, and was promising for the development of drug delivery systems. The interstitial hollow cavities served as depots to accommodate various therapeutic agents, and mesopores enabled therapeutic agents to diffuse through the shell. Furthermore, the surface silanol groups on the mesopores and external surface enabled versatile and selective functionalization for anchoring targeting (e.g. DNA aptamer LXL-1) or functional (e.g. photosensitizer PpIX) moieties. In short, we developed a novel nano combination therapeutic approach that targeted TNBC. The combination of PDT and TPZ eradicated cancer cells synergistically and effectively in both normoxic and hypoxic regions of tumor tissues. This nanotherapy enhanced the retainment of chemotherapy drugs in tumors, yet decreased drug accumulation in the other non-target organs, which suggested it is a promising strategy for treating TNBC. Our study not only verified the feasibility of PDT/BD combination therapy in cancer treatment, but also paved the way for the development of a therapeutic strategy for malignant neoplasm in hypoxic regions.

## Conclusion

Given the lack of effective treatments for TNBC, numerous efforts have been devoted in the past to augment therapeutic opportunities for TNBC patients. The phase III IMpassion130 trial using chemotherapy plus atezolizumab (a fully humanized, engineered monoclonal antibody of IgG1 against the programmed cell death ligand 1, PD-L1) compared with chemotherapy plus placebo brought breast cancer into the era of antibody-based therapeutic approaches; however, limitations of the therapeutic antibody approach included high medical cost, poor tissue accessibility, insufficient pharmacokinetics, and imperfect interactions with the immune system. Previous studies have been reported on the applicability of nanoDrug Delivery Systems; however, the effectiveness of PDT/BD combination nanotherapy in tumor hypoxia was less frequently discussed. Herein, we successfully developed a synergistic approach to target TNBC under both normoxia and hypoxic conditions. The use of HMSNs modified with the aptamer, LXL-1, was confirmed to target TNBC and release TPZ to eradicate tumors under hypoxic conditions. On the other hand, a photosensitizer that was fixed inside HMSNs generated a sufficient level of radicals to shrink tumors under normoxic conditions with PDT. This design employed the mechanism of action using a combination of two medicines, which demonstrated promising potential for TNBC therapy. These observations encourage us to conduct further investigations of our nanoVector to treat hypoxia-associated diseases because hypoxia-induce heterogeneous environments promote tumor invasiveness, angiogenesis, drug resistance, and metastasis, and impair therapeutic efficacy.

## Methods

### ***Chemicals and reagents***

All chemicals and reagents were of analytical grade and were used as received without further purification. Benzylcetyldimethyl-ammonium chloride (BCDAC, ≥97%), bovine serum albumin (BSA), diethylene glycol hexadecyl ether (C16E2, ≥95%), 2',7'-dichlorodihydrofluorescein diacetate, 1,3 diphenylisobenofuran, L-glutamine, paraformaldehyde, potassium chloride (KCl), 2-(N-Morpholino)ethanesulfonic acid (MES), N-Succinimidyl 4-(maleimidomethyl)cyclohexanecarboxylate (SMCC), potassium iodide, potassium phosphate monobasic, potassium phosphate dibasic, propidium iodide, PpIX, sodium chloride, sodium hydroxide (NaOH), sodium pyruvate, thiazolyl blue tetrazolium bromide, tirapazamine (TPZ), tris(2-carboxyethyl)phosphine (TCEP), 1-ethyl-3-(3-dimethylaminopropyl)-carbodiimide (EDC), N-hydroxysuccinimide (NHS), ethanol, hydrochloric acid (HCl), trypan blue solution, and trypsin were obtained from Sigma-Aldrich (St. Louis, MO, USA). Aminopropyltrimethoxysilane (APTMS) and tetramethoxysilane (TEOS) was purchased from Acros Organics (Morris, New Jersey, USA). Acetone, dimethyl sulfoxide (DMSO), sodium dodecyl sulfate (SDS), and tris base were obtained from J.T. Baker (Phillipsburg, New Jersey, USA). Dulbecco's Modified Eagles Medium (DMEM) media (high glucose), Fetal Bovine Serum (FBS), and penicillin-streptomycin were acquired from Life Technologies (Carlsbad, CA, USA). Thiolated DNA aptamer LXL-1 was obtained from Integrated DNA Technologies (Coralville, IA, USA). Deionized distilled water (18.2 MΩ·cm) was acquired from Milli-Q system (Milford, MA, USA) and used for all aqueous solution preparations.

### ***Preparation of TPZ@LXL-1-PpIX-MMT-2***

MMT-2 was synthesized following the procedures reported previously [33]. In a typical synthesis, TEOS (5.98 mL) was injected to the solution that contained BCDAC (0.74 g), C16E2 (0.26 g), 0.4 M NaOH (21.4 mL), and water (575 mL) at a rate of 7.5 mL $h^{-1}$ at 35 $^{o}$C. The mixture was stirred at 35 $^{o}$C for 2 h and

then was aged at 90 °C for 24 h. The solid product was filtered, washed with water and acetone, and finally dried at 60 °C.

The external surface of MMT-2 was first functionalized by maleimide group for later conjugation with LXL-1. The dehydrated as-synthesized MMT-2 (400 mg), was stirred into a solution that contained 0.5 mL APTMS and 40 mL toluene at 80 °C for 12 h. The solid was filtered and washed with ethanol. The surfactant was then removed by repetitive extraction with HCl-ethanol solution, and the resulting sample that was referred to as $NH_2$-MMT-2. $NH_2$-MMT-2 (250 mg) was dispersed in 10 mM PBS (20 mL), which was followed by mixing with sulfo-SMCC (25 mg dissolved in 5 mL of 40% DMSO in PBS (v/v, 10 mM, pH 7.4). After stirring at 25 °C for 0.5 h, the resulting solid with maleimide groups on the external surface (designated as M-MMT-2) was filtered and washed repeatedly with water and acetone.

Next, PpIX was modified on the mesopores of MMT-2. The dehydrated M-MMT-2 was stirred in a solution that contained 0.5 mL APTMS and 25 mL toluene at 80 °C 12 h. The resulting solid, which was referred to as M-$NH_2$-MMT-2, was filtered, washed by ethanol, and dried at 60 °C. Subsequently, M-$NH_2$-MMT-2 was dispersed in 25 mM 2-(N-morpholino) ethanesulfonic acid (MES) buffer (20 mL, pH 6.1), and 1-ethyl-3-(3-dimethylaminopropyl)-carbodiimide (EDC, 25.0 mg), N-hydroxysuccinimide (NHS, 27.5 mg), and PpIX (25 mg dissolved in 5 mL of 40% ethanol in MES buffer (v/v, 25 mM, pH 6.1) were introduced to the dispersion. The mixture was stirred at 35 °C for 24 h, and the solid product was filtered and washed repeatedly by water and acetone and finally dried overnight under vacuum. The resulting sample that contained PpIX was referred to as M-PpIX-MMT-2.

Finally, DNA aptamer LXL-1 was conjugated with maleimide group on the external surface of MMT-2 through a thiol-ene reaction. Prior to the reaction, the thiolated LXL-1 (10 µL, 200 µM) was mixed with Tris(2-carboxyethyl)phosphine (TCEP, 3.4 µL, 1 mM) at room temperature for 30 min to ensure the reduction of disulfide bonds. M-PpIX-MMT-2 (0.5 mg) was then added, and the mixture was allowed to stir at room temperature for 4 h in PBS (pH 7.4, 1 mM, 500 µL). The solid was centrifuged (9500 × g), followed by washing with distilled, deionized water. After subsequent centrifugation, the

resulting sample, which was referred to as LXL-1-PpIX-MMT-2, was collected and dried at 37 $^oC$.

LXL-1-PpIX-MMT-2 was loaded with TPZ by repeated incipient wetness impregnation using an aqueous solution of TPZ (1.2 mg/mL in deionized water). The sample was dried at 37 $^oC$ after every impregnation step. The molar ratio of TPZ and PpIX in the resulting nanoVector TPZ@LXL-1-PpIX-MMT-2 was ~150.

***Characterization of materials***

XRD patterns were recorded on a Mac Science 18MPX diffractometer (Tokyo, Japan), using Cu K$\alpha$ radiation. TEM images were obtained using a JEOL JEM-2010 microscope (Tokyo, Japan) that operated at 200 kV. TGA was carried out in air using a Mettler-Toledo TGA/DSC 2-HT device (Leicester, UK). FTIR spectra were analyzed using the Bruker Tensor 27 device (Manning Park Billerica, MA, USA). A Varian Cary 300 UV–Vis Bio spectrophotometer (Palo Alto, CA, USA) was used for quantitative determination of PpIX. A Zetasizer Nano ZS (Malvern Instruments, UK) was used to determine the hydrodynamic size of nanomaterials by DLS, and their zeta potentials were determined using laser doppler microelectrophoresis. Prior to size and zeta potential measurements, particles were dispersed in PBS (pH 7.4) at a concentration of 0.01 mg/mL followed by sonication for 3 min.

***Cell culture***

MDA-MB-231 (ATCC, HTB-26™), MCF-7 (ATCC, HTB-22™) human breast cancer cell lines, and MCF-10A (ATCC, CRL-10317™) human breast epithelial cell lines that were obtained from the Bioresource Collection and Research Center (BCRC) (Hsinchu, Taiwan) were tested in this study. MDA-MB-231, MCF-7 and MCF-10A cells were cultured with DMEM media, supplemented with 10% FBS, sodium pyruvate (100 mg/L), L-glutamine (550 mg/L), and 1% penicillin-streptomycin in a humidified incubation system that contained 5% $CO_2$ at 37$^oC$. The growth medium was changed every 48 h, and cells were trypsinized (using 0.1% trypsin) and subcultured when they grew to about 90% confluence.

***Cellular uptake of PpIX (free PpIX compared with LXL-1-PpIX-MMT-2) and in vivo targeting***

To test the selectivity of LXL-1-PpIX-MMT-2 toward MDA-MB-231, MCF-7, and MCF-10, cells were first incubated in confocal dishes for 18 h and then treated with 1.17 μg/mL of LXL-1-PpIX-MMT-2 (The amount of PpIX conjugated on LXL-1-PpIX-MMT-2 was equivalent to 0.4 μM free PpIX); after 5 h of co-incubation, the cells were rinsed two times with PBS, fixed with 4% paraformaldehyde for 5 min. Cells were then photographed under a confocal microscope (Observer Z1, Zeiss, Germany) with a 63x oil immersion objective (Plan-Apochromat 63x/1.40 Oil DIC M27, Zeiss, Germany)

To determine the delivery efficiency of LXL-1-PpIX-MMT-2, the intracellular PpIX level among three cell lines, MDA-MB-231, MCF-7, and MCF-10A, treated with either 0.4 μM of free PpIX or 1.17 μg/mL of LXL-1-PpIX-MMT-2 (The amount of PpIX conjugated on LXL-1-PpIX-MMT-2 was equivalent to 0.4 μM free PpIX), was measured. After co-incubation for 5 h, the suspension was removed, and the cells were washed twice with PBS, followed by trypsinization. The isolated cells were mixed with the RIPA lysis buffer to release the cellular content. After 15 min of vortexing, the resulting solution was centrifuged (21,380 x g, 5 min). The supernatant of the cell extract was then transferred to a 96-well black plate and subjected to fluorometric analysis using a spectrophotometer ($\lambda_{ex}$ = 401 nm, $\lambda_{em}$ = 635 nm). The internalized PpIX was quantified through a standard calibration curve, which was obtained after adding a series of known concentrations of PpIX to the extraction solution. In addition, the protein concentration of the cell lysates was also determined using the Bradford protein assay. The protein amount was used to normalize the obtained results for each measurement to eliminate the fluctuation due to the slightly different number of cells per well.

All animal experiments conducted in current study were performed in compliance with the NHMRC Taiwan Code of Practice for the care and use of animals for scientific purposes. Female NU/NU nude mice (4 wk old) were purchased from the Biolasco Animal Feeding Farm (Taipei, Taiwan). To investigate the *in vivo* targeting efficiency of our nanoVectors, subcutaneous human xenograft tumor

models in nude mice were established after 10 d of animal stabilization through inoculation of MDA-MB-231 at a density of ca. $5\times10^6$ (suspended in 100 μL sterile physiological saline solution) on two thighs. Tumor growth was allowed to proceed when tumor volume reached the size of 15 mm. Tumor-bearing animals were given an intraperitoneal injection (i.p) with LXL-1-PpIX-MMT-2 (5.2 mg/mL) at a dose of 100 µg of PpIX (volume of injection: 100 μL, HMSN concentration: 5.2 mg/mL). After 6 h, the mice were sacrificed, and the tissues were immediately removed, which included heart, liver, spleen, lung, kidney, and the tumor. The *In Vivo* Imaging System (IVIS, PerkinElmer, Branford, CT, USA) was then used to determine the in vivo targeting efficiency of LXL-1-PpIX-MMT-2 presented in the tumor.

***In vitro effectiveness of PpIX, TPZ, and*** **TPZ@*LXL-1-PpIX-MMT-2***

To investigate the *in vitro* effectiveness of PpIX, TPZ, and nanoVectors, cell viability studies at both normoxia and hypoxia condition were conducted. For normoxia effectiveness, MDA-MB-231 cells were first seeded in a 96-well plate at a density of $10^4$ per well/well and then placed directly in a cell incubator for 18 h after addition of designated drugs. To test for hypoxia effectiveness, home-made double-layered atmosphere bags (atmobag) filled with the desired gas composition, which was $N_2$ 5% $CO_2$, and oxygen levels of 5%, 2%, or 1%, were used to mimic the hypoxic condition experimentally. We treated tested cells with TPZ@LXL-1-PpIX-MMT-2 that was suspended in PBS originally and mixed with culture medium at an appropriate ratio prior to use, or appropriate concentration of free PpIX, which was dissolved in DMSO originally and mixed with culture medium at a ratio of 1:99 prior to use. The dosage of PpIX used to treat the cells ranged from 0 to 0.8 μM (equivalent to 0–0.46 µg/mL), and 0–80 µM (equivalent to 0–14.67 µg/mL) was employed for free TPZ. For photoactivation of PpIX, LED light irradiation ($\lambda$= 630 nm; power: 15.15 mW; fluence: 5.73 x $10^{20}$ $m^{-2}s^{-1}$; applied time: 0 to 4 min, photo illustration of the light source was shown in **Figure S5**) was applied 5 h after dosing with PpIX, which was followed by placing the cells in the atmobag with the preferable oxygen level and then incubating for an additional 19 h.

***Therapeutic effectiveness of TPZ@LXL-1-PpIX-MMT-2 using a MDA-MB-231 xenograft tumor model***

To study the *in vivo* therapeutic effectiveness of TPZ@LXL-1-PpIX-MMT-2, tumor-bearing animals were subjected to intratumoral injections (i.t.) of TPZ@LXL-1-PpIX-MMT-2 (5.2 mg/mL) at a dose of 100 µg of PpIX (volume of injection: 100 µg of PpIX, HMSN concentration: 5.2 mg/mL)) on the 22$^{nd}$ and 29$^{th}$ days. Five different groups of mice were evaluated, which included (A) Control group: injection of 100 µL sterile PBS; (B) PpIX-alone group: injection of 100 µL of 1 mg/mL free PpIX; (C) TPZ-alone group: infusion of 100 µL of 0.5 mg/mL free TPZ; (D) Combinatorial therapy group: infusion of 100 µL sterile solution that contained 1 mg/mL PpIX and 0.5 mg/mL TPZ; (E) NanoVector group: infusion of 100 µL the nanoVector suspension, TPZ@LXL-1-PpIX-MMT-2, at a concentration of 5.2 mg/mL. Immediately after administration of therapeutic agents, the tumors were treated with 10 min of 630 nm LED irradiation. After phototherapeutic treatment, the experimental animals were allowed to recover. The body weight of experimental animals was recorded, and sizes of all tumors were monitored by estimating V (volume) = length × width$^2$ × 0.5 for the entire study period. Each group was subjected to the exposure of LED irradiation once. Finally, on the 36$^{th}$ day, mice were sacrificed and tumors were removed.

***Histological analysis of tumors***

For histological studies, tumor tissue was fixed in 10% formalin for one week and embedded in paraffin. Tumor tissue was sectioned (3 µm) before being fixed on glass slides and allowed to dehydrate overnight. Sections were subjected to the dewaxing and rehydration through the use of xylene and a series of decreasing alcohol concentrations (100%, 95%, 90%, 80% ethanol/dd$H_2O$, and finally dd$H_2O$). For hematoxylin and eosin stain (H&E) analysis, sections were stained with hematoxylin and eosin to confirm the cell density and to observe the details of cellular and tissue structures. To visualize hypoxic areas immunohistochemically, a commercially available hypoxyprobe kit (Hypoxyprobe™-1 Omni kit, Hypoxyprobe, USA) was used according to the manufacturer's protocol. In brief, each group of animals

was i.p. administered with 60 mg/kg Hypoxyprobe™-1 solution (Pimonidazole) 1 h before sacrifice; the tumor tissue that was intended to be analyzed for the amount of hypoxia was prepared as described above. Next, the deparaffinized tissue sections were treated with 3% $H_2O_2$ to block endogenous peroxidase activity, followed by incubation with FBS to reduce non-specific binding. The primary antibody (PAb2627A) (1:200, Hypoxyprobe, Inc, USA) was added to the tissue section-mounted slide and allowed to react overnight at 4 °C. After washing three times with Tris-buffered Saline (TBS) with tween-20, the slide was subsequently incubated with the secondary antibody for 1 h to complete tissue preparation for immunostaining.

***Statistical analysis***

Experiments were performed in triplicate and repeated at least three times. Data were presented as means ± standard deviation (SD). The *t*-test was used to evaluate whether there was any statistical significance between the means of two independent groups. In this study, p-values of <0.05 represented results that were statistically significant, and p-values of <0.01 were considered to be highly statistically significant.

## Acknowledgements

The authors gratefully acknowledge the financial support from the Ministry of Education of Taiwan (Aim for Top University Program) and the Ministry of Science and Technology (MOST) of Taiwan, under the contracts Nos. MOST 102-2628-M-002-004-MY4, 106-2113-M-002-014-MY3, 106-2113-M-007-025-MY3, 107-3017-F-007-002 and MOST 107-2113-M-260-002-MY2. This study was also supported by the Frontier Research Center on Fundamental and Applied Sciences of Matters from The Featured Areas Research Center Program within the framework of the Higher Education Sprout Project by the Ministry of Education (MOE) in Taiwan. *In Vivo* Imaging work was performed at the Instrumentation Center of in University; TGA experiments were conducted at the Instrumentation Centers of National Tsing Hua University.

## Supplementary information

Supplementary information accompanies this paper at https ://

Additional file 1: **Figure S1** XRD pattern of MMT-2. **Figure S2.** The cytotoxicity of two mesoporous silica nanoparticles (MMT-2, and LXL-1-MMT-2) on MDA-MB-231 cells. Various concentrations of nanoVector (0, 1.12, 2.24, 4.48, 8.96, and 17.92 g/mL) were used to treat cells. Cell viability was assessed after 24 h of treatment. Each bar represents an average of at least three repetitive analyses. **Figure S3.** Quantitative data of singlet oxygen molecules generated by photoexcitation of PpIX. The singlet oxygen molecules generated by 630 nm of LED in a DPBF quantitative PpIX. (a) The concentrations of PpIX were 0, 0.2, 0.4 and 0.8 µM, and the irradiation times were 10, 20, and 30 sec. (b) The oxygen concentrations were 0%, 1%, 5%, and 21%, the PpIX concentration was 0.8 µM, and the irradiation time was 10, 20, and 30 sec. (c) DPBF concentration of 250 µM, PpIX concentration of 0.4

μM, PpIX-MMT2 concentration of 1.17 g/mL, LXL-1-PpIX-MMT-2 concentration of 1.17 g/mL, and irradiation times of 0, 10, 20, 30, 40, and 50 sec. Each data point represented an average of three replicates. **Figure S4.** Quantification of ROS produced by cells irradiated with PpIX at different oxygen concentrations with different lengths of time of irradiation. The concentration of PpIX was 0.4 μM, DCFDA concentration was 40 μM, and the irradiation times were 0, 1, 2, 3, and 4 min. All data represented an average of three replicates. **Figure S5.** Photo illustration of LED light sources. (a) LED light device that was suitable for use in 96 microplate format. (b) The irradiation of cells on a 96-well plate was achieved by stacking the LED light device (as seen in a) and black 96-well plate (for guiding light) on the white plate cultured with various groups of cells (c) The LED device that was used in animal studies.

## Authors' contributions

YTC, CYL and JWW contributed equally to this work. CMY's group was responsible for the synthesis, functionalization and characterization of thin-shell hollow mesoporous Ia3d silica nanoparticles; JAH's helped with surface modification of nanoparticles, and was responsible for *in vitro* and *in vivo* studies with L.C.W.'s group. L.C.H. provided technical help for *in vivo* imaging. All authors participated in the writing of the manuscript. All authors discussed the results and implications and commented on the manuscript at all stages.

## Competing interests

The authors declare that they have no known competing financial interests or personal relationships that could have appeared to influence the work reported in this paper.

## Ethics approval and consent to participate

All animal experiments conducted in current study were performed in compliance with the NHMRC Taiwan Code of Practice for the care and use of animals for scientific purposes, and approved by the institutional animal care and use committee (IACUC) of National Taiwan University.

## Consent for publication

Not applicable.

## Availability of data and material

All data generated or analyzed during this study are included in this manuscript.

## Funding

The authors gratefully acknowledge the financial support from the Ministry of Education of Taiwan (Aim for Top University Program) and the Ministry of Science and Technology (MOST) of Taiwan, under the contracts Nos. MOST 102-2628-M-002-004-MY4, 106-2113-M-002-014-MY3, 106-2113-M-007-025-MY3, 107-3017-F-007-002 and MOST 107-2113-M-260-002-MY2. This study was also supported by the Center for Emerging Materials and Advance Devices of National Taiwan University, and Frontier Research Center on Fundamental and Applied Sciences of Matters from The Featured Areas Research Center Program within the framework of the Higher Education Sprout Project by the Ministry of Education (MOE) in Taiwan.

## Author details

[1]BioAnalytical Chemistry and Nanobiomedicine Laboratory, Department of Biochemical Science and Technology, National Taiwan University, Taipei 10617, Taiwan; [2]Department of Chemistry, National Tsing Hua University, Hsinchu 30013, Taiwan; [3]NTU Instrumentation Center, Technology Commons, College of Life Science, National Taiwan; University, Taipei 10617, Taiwan; [4]Frontier Research Center

on Fundamental and Applied Sciences of Matters, National Tsing Hua University, Hsinchu 30013, Taiwan; [5]Department of Applied Chemistry, National Chi Nan University, Puli, Nantou 54561, Taiwan; [6]Biotechnology Center, National Taiwan University, Taipei 10617, Taiwan; [7]Department of Chemistry, National Taiwan University, Taipei 10617, Taiwan

## References


1. Siegel RL, Miller KD, Jemal A. **Cancer statistics, 2020.** *CA Cancer J Clin* 2020, **70:**7-30.
2. Chavez KJ, Garimella SV, Lipkowitz S. **Triple negative breast cancer cell lines: one tool in the search for better treatment of triple negative breast cancer.** *Breast Dis* 2010, **32:**35-48.
3. Haffty BG, Yang Q, Reiss M, Kearney T, Higgins SA, Weidhaas J, Harris L, Hait W, Toppmeyer D. **Locoregional relapse and distant metastasis in conservatively managed triple negative early-stage breast cancer.** *J Clin Oncol* 2006, **24:**5652-5657.
4. Foulkes WD, Smith IE, Reis-Filho JS. **Triple-negative breast cancer.** *N Engl J Med* 2010, **363:**1938-1948.
5. Trudeau M, Charbonneau F, Gelmon K, Laing K, Latreille J, Mackey J, McLeod D, Pritchard K, Provencher L, Verma S. **Selection of adjuvant chemotherapy for treatment of node-positive breast cancer.** *Lancet Oncol* 2005, **6:**886-898.
6. Ma F, Li H, Wang H, Shi X, Fan Y, Ding X, Lin C, Zhan Q, Qian H, Xu B. **Enriched CD44(+)/CD24(-) population drives the aggressive phenotypes presented in triple-negative breast cancer (TNBC).** *Cancer Lett* 2014, **353:**153-159.
7. Li H, Ma F, Wang H, Lin C, Fan Y, Zhang X, Qian H, Xu B. **Stem cell marker aldehyde dehydrogenase 1 (ALDH1)-expressing cells are enriched in triple-negative breast cancer.** *Int J Biol Markers* 2013, **28:**e357-364.
8. Honeth G, Bendahl PO, Ringner M, Saal LH, Gruvberger-Saal SK, Lovgren K, Grabau D, Ferno M, Borg A, Hegardt C. **The CD44(+)/CD24(-) phenotype is enriched in basal-like breast tumors.** *Breast Cancer Research* 2008, **10**.
9. Jhan JR, Andrechek ER. **Triple-negative breast cancer and the potential for targeted therapy.** *Pharmacogenomics* 2017, **18:**1595-1609.
10. Costa RLB, Han HS, Gradishar WJ. **Targeting the PI3K/AKT/mTOR pathway in triple-negative breast cancer: a review.** *Breast Cancer Res Treat* 2018, **169:**397-406.
11. Liu CC, Prior J, Piwnica-Worms D, Bu GJ. **LRP6 overexpression defines a class of breast cancer subtype and is a target for therapy.** *Proceedings of the National Academy of Sciences of the United States of America* 2010, **107:**5136-5141.
12. Tung NM, Garber JE. **BRCA1/2 testing: therapeutic implications for breast cancer management.** *Br J Cancer* 2018, **119:**141-152.
13. Ssempala A. **Vaccine targeting HIF1A in triple negative breast cancer.** *European Journal of Cancer* 2017, **72:**S26-S26.
14. McKeown SR. **Defining normoxia, physoxia and hypoxia in tumours-implications for treatment response.** *Br J Radiol* 2014, **87:**20130676.
15. Agostinis P, Berg K, Cengel KA, Foster TH, Girotti AW, Gollnick SO, Hahn SM, Hamblin MR, Juzeniene A, Kessel D, et al. **Photodynamic therapy of cancer: an update.** *CA Cancer J Clin* 2011, **61:**250-281.
16. Detty MR, Gibson SL, Wagner SJ. **Current clinical and preclinical photosensitizers for use in photodynamic therapy.** *J Med Chem* 2004, **47:**3897-3915.
17. Dolmans DE, Fukumura D, Jain RK. **Photodynamic therapy for cancer.** *Nat Rev Cancer* 2003,

**3:**380-387.

18. Banerjee SM, MacRobert AJ, Mosse CA, Periera B, Bown SG, Keshtgar MRS. **Photodynamic therapy: Inception to application in breast cancer.** *Breast* 2017, **31:**105-113.
19. Dang J, He H, Chen D, Yin L. **Manipulating tumor hypoxia toward enhanced photodynamic therapy (PDT).** *Biomater Sci* 2017, **5:**1500-1511.
20. Muz B, de la Puente P, Azab F, Azab AK. **The role of hypoxia in cancer progression, angiogenesis, metastasis, and resistance to therapy.** *Hypoxia (Auckl)* 2015, **3:**83-92.
21. Graham K, Unger E. **Overcoming tumor hypoxia as a barrier to radiotherapy, chemotherapy and immunotherapy in cancer treatment.** *International Journal of Nanomedicine* 2018, **13:**6049-6058.
22. Vakrat-Haglili Y, Weiner L, Brumfeld V, Brandis A, Salomon Y, McLlroy B, Wilson BC, Pawlak A, Rozanowska M, Sarna T, Scherz A. **The microenvironment effect on the generation of reactive oxygen species by Pd-bacteriopheophorbide.** *J Am Chem Soc* 2005, **127:**6487-6497.
23. Shen S, Zhu CL, Huo D, Yang MX, Xue JJ, Xia YN. **A Hybrid Nanomaterial for the Controlled Generation of Free Radicals and Oxidative Destruction of Hypoxic Cancer Cells.** *Angewandte Chemie-International Edition* 2017, **56:**8801-8804.
24. Siim BG, Pruijn FB, Sturman JR, Hogg A, Hay MP, Brown JM, Wilson WR. **Selective potentiation of the hypoxic cytotoxicity of tirapazamine by its 1-N-oxide metabolite SR 4317.** *Cancer Res* 2004, **64:**736-742.
25. Reddy SB, Williamson SK. **Tirapazamine: a novel agent targeting hypoxic tumor cells.** *Expert Opin Investig Drugs* 2009, **18:**77-87.
26. Zeman EM, Brown JM, Lemmon MJ, Hirst VK, Lee WW. **SR-4233: a new bioreductive agent with high selective toxicity for hypoxic mammalian cells.** *Int J Radiat Oncol Biol Phys* 1986, **12:**1239-1242.
27. Junnotula V, Sarkar U, Sinha S, Gates KS. **Initiation of DNA strand cleavage by 1,2,4-benzotriazine 1,4-dioxide antitumor agents: mechanistic insight from studies of 3-methyl-1,2,4-benzotriazine 1,4-dioxide.** *J Am Chem Soc* 2009, **131:**1015-1024.
28. Li Z, Barnes JC, Bosoy A, Stoddart JF, Zink JI. **Mesoporous silica nanoparticles in biomedical applications.** *Chem Soc Rev* 2012, **41:**2590-2605.
29. Trewyn BG, Slowing, II, Giri S, Chen HT, Lin VS. **Synthesis and functionalization of a mesoporous silica nanoparticle based on the sol-gel process and applications in controlled release.** *Acc Chem Res* 2007, **40:**846-853.
30. Wu SH, Hung Y, Mou CY. **Mesoporous silica nanoparticles as nanocarriers.** *Chemical Communications* 2011, **47:**9972-9985.
31. Rosenholm JM, Mamaeva V, Sahlgren C, Linden M. **Nanoparticles in targeted cancer therapy: mesoporous silica nanoparticles entering preclinical development stage.** *Nanomedicine* 2012, **7:**111-120.
32. He QJ, Shi JL. **Mesoporous silica nanoparticle based nano drug delivery systems: synthesis, controlled drug release and delivery, pharmacokinetics and biocompatibility.** *Journal of Materials Chemistry* 2011, **21:**5845-5855.

33. Lai NC, Lin CH, Ku PH, Chang LL, Liao KW, Lin WT, Yang CM. **Hollow mesoporous Ia3d silica nanospheres with singleunit-cell-thick shell: Spontaneous formation and drug delivery application.** *Nano Research* 2014, **7:**1439-1448.
34. Tu HL, Lin YS, Lin HY, Hung Y, Lo LW, Chen YF, Mou CY. **In vitro Studies of Functionalized Mesoporous Silica Nanoparticles for Photodynamic Therapy.** *Advanced Materials* 2009, **21:**172-+.
35. Sachar M, Anderson KE, Ma X. **Protoporphyrin IX: the Good, the Bad, and the Ugly.** *J Pharmacol Exp Ther* 2016, **356:**267-275.
36. Teng IT, Chang YJ, Wang LS, Lu HY, Wu LC, Yang CM, Chiu CC, Yang CH, Hsu SL, Ho JA. **Phospholipid-functionalized mesoporous silica nanocarriers for selective photodynamic therapy of cancer.** *Biomaterials* 2013, **34:**7462-7470.
37. Adams GE, Stratford IJ. **Bioreductive drugs for cancer therapy: the search for tumor specificity.** *Int J Radiat Oncol Biol Phys* 1994, **29:**231-238.
38. Baas P, Oppelaar H, Stavenuiter M, Vanzandwijk N, Stewart FA. **Interaction of the Bioreductive Drug Sr 4233 and Photodynamic Therapy Using Photofrin in a Mouse-Tumor Model.** *International Journal of Radiation Oncology Biology Physics* 1993, **27:**665-670.
39. Hao JQ, Li Q, Xu SP, Shen YX, Sun GY. **Effect of lumiracoxib on proliferation and apoptosis of human nonsmall cell lung cancer cells in vitro.** *Chinese Medical Journal* 2008, **121:**602-607.
40. Liu Y, Liu Y, Bu W, Cheng C, Zuo C, Xiao Q, Sun Y, Ni D, Zhang C, Liu J, Shi J. **Hypoxia Induced by Upconversion-Based Photodynamic Therapy: Towards Highly Effective Synergistic Bioreductive Therapy in Tumors.** *Angew Chem Int Ed Engl* 2015, **54:**8105-8109.
41. Broekgaarden M, Weijer R, van Wijk AC, Cox RC, Egmond MR, Hoebe R, van Gulik TM, Heger M. **Photodynamic Therapy with Liposomal Zinc Phthalocyanine and Tirapazamine Increases Tumor Cell Death via DNA Damage.** *J Biomed Nanotechnol* 2017, **13:**204-220.
42. Turner N, Moretti E, Siclari O, Migliaccio I, Santarpia L, D'Incalci M, Piccolo S, Veronesi A, Zambelli A, Del Sal G, Di Leo A. **Targeting triple negative breast cancer: Is p53 the answer?** *Cancer Treatment Reviews* 2013, **39:**541-550.
43. Na B, Yu X, Withers T, Gilleran J, Yao M, Foo TK, Chen CX, Moore D, Linz Y, Kimball SD, et al. **Therapeutic targeting of BRCA1 and TP53 mutant breast cancer through mutant p53 reactivation.** *Npj Breast Cancer* 2019, **5**.
44. Le QT, Moon J, Redman M, Williamson SK, Lara PN, Jr., Goldberg Z, Gaspar LE, Crowley JJ, Moore DF, Jr., Gandara DR. **Phase II study of tirapazamine, cisplatin, and etoposide and concurrent thoracic radiotherapy for limited-stage small-cell lung cancer: SWOG 0222.** *J Clin Oncol* 2009, **27:**3014-3019.
45. Patel SN, Mango VL, Jadeja P, Friedlander L, Desperito E, Wynn R, Feldman S, Ha R. **Reflector-guided breast tumor localization versus wire localization for lumpectomies: A comparison of surgical outcomes.** *Clinical Imaging* 2018, **47:**14-17.
46. Zoon CK, Starker EQ, Wilson AM, Emmert-Buck MR, Libutti SK, Tangrea MA. **Current molecular diagnostics of breast cancer and the potential incorporation of microRNA.** *Expert Review of Molecular Diagnostics* 2009, **9:**455-467.

## Figure Caption

**Scheme 1.** Schematic representation of the nanoVector fabrication and the nanotherapeutic strategy for treating Triple Negative Breast Cancer (TNBC) Cells. (a) Illustration of the nanoVector fabrication. (b) Illustration of antitumor activity of the novel PDT/BD combination nanotherapeutic strategy. The hollow mesoporous silica nanoparticles (HMSN MMT-2) was used as a vector, which was functionalized with PS (PpIX) and loaded with bioreductive drugs (TPZ). The surface of MMT-2 was further modified with the DNA aptamer, LXL-1, which resulted in the targeted drug delivery system that selectively targeted TNBC cell line, MDA-MB-231. Oxygen consumption caused by the irradiation of PpIX led to the activation of TPZ and enhanced antitumor activity, which resulted in the synergism of PDT and bioreductive chemotherapy

**Figure 1.** Characterization of HMSN. (a,b) Typical Transmission electron microscopic (TEM) images of MMT-2 (a) and LXL-1-PpIX-MMT-2 (b). (c) Thermogravimetric analysis (TGA) data of M-MMT-2 and M-PpIX-MMT-2. (d) Fourier-transform infrared spectroscopic (FTIR) spectra of MMT-2, M-MMT-2, and M-PpIX-MMT-2. Assignment of the IR peaks: (i) N-H stretching of secondary amine; (ii) C-H stretching of alkanes; (iii) C=O stretching; (iv) N-H bending of amide; (v) C-H bending of alkanes; (vi) C=C bending. (e) Zetasizer confirmed the size and zeta potential of our MSN nanoparticles using the techniques of Dynamic Light Scattering (DLS) and Electrophoretic Light Scattering (ELS).

**Figure 2.** The *in vitro/in vivo* targeting of LXL-1 aptamer toward TNBC cells, MDA-MB-231. *In vitro* study was done by treating TNBC with 0.4 μM of PpIX or 1.17 μg/mL of LXL-1-PpIX-MMT-2 (The amount of PpIX conjugated on LXL-1-PpIX-MMT-2 was equivalent to 0.4 μM free PpIX), and was allowed to incubate for 5 h. No treatment was received by control group. (a) Quantification of the intracellular PpIX in MDA-MB-231 cell groups treated with either free PpIX or LXL-1-PpIX-MMT-2 and normalized by total cellular protein (nmol PpIX/mg protein). (b) Intracellular distribution of free PpIX or LXL-1-PpIX-MMT-2 in MDA-MB-231 cells observed under confocal microscopy (Scale bar represents 10 μm). (c) Quantification of the intracellular PpIX in various breast cancer cells (MDA-MB-231, MCF-7, MCF-10A) treated with LXL-1-PpIX-MMT-2 and normalized by total cellular protein (nmol PpIX/mg protein). (d) Investigation of intracellular distribution of LXL-1-PpIX-MMT-2 in MDA-MB-231, MCF-7, and MCF-10A by confocal microscopy (Scale bar represents 20 μm). All data represent average values of at least three replicates, and the error bars reflect standard deviation. (e) *In vivo* targeting of the nanoVector, LXL-1-PpIX-MMT-2, in a TNBC xenografted tumor model. TNBC were inoculated in NU/NU female mice. After the tumors reached a palpable size of 15 mm, 100 μg of PpIX was injected intraperitoneally into experimental animals. The organs (heart, liver, spleen, lung, kidney, tumor) were taken out 6 h after injection and the fluorescence intensity of PpIX was measured using IVIS.

**Figure 3.** Effects of drug dosage, oxygen level, and photoirradiation time on cell viabilities of TNBC cells, MDA-MB-231. (a) Cell viabilities of MDA-MB-231 treated with various concentrations of PpIX (0, 0.2, 0.4, 0.8 μM) under 21% $O_2$ for different photoirradiation times (0, 1, 2, 3, 4 min). (b) Cell viabilities of MDA-MB-231 treated with various concentrations of PpIX under 5% $O_2$ for different photoirradiation times. (c) Cell viabilities of MDA-MB-231 treated with various concentrations of PpIX under 2% $O_2$ for different photoirradiation times. (d) Cell viabilities of MDA-MB-231 treated with various concentrations of PpIX under 1% $O_2$ for different photoirradiation times. (e) Cell viabilities of MDA-MB-

231 treated with various concentrations of TPZ (0, 20, 60, 100 μM) under different oxygen levels (21%, 5%, 2%, 1%). MTT assay was used to confirm cell survival after 24 h.

**Figure 4.** The cytotoxic effect of nanoVector, TPZ@LXL-1-PpIX-MMT-2, under hypoxia condition. (a) Cell viabilities of MDA-MB-231 treated with 0.4 μM of PpIX, 60 μM of TPZ, PpIX+TPZ, and TPZ@LXL-1-PpIX-MMT-2 under various oxygen levels (5%, 2%, 1%). Photoirradiation was performed 5 h after treatment, and the irradiation time was 1 min. No treatment was received by control group. (b) Coefficient of Drug Interaction (CDI) of various chemotherapeutic treatments for TNBC cells. MTT assay was conducted to determine the viability 24 h after treatment. All experiments were performed at least in triplicate; all data are expressed as the mean ± standard deviation.

**Figure 5.** *In vivo* antitumor efficacy of the nanoVector, TPZ@LXL-1-PpIX-MMT-2. Human MDA-MB-231 cells were inoculated in NU/NU female mice. After the tumors reached a palpable size of 5 mm, various formats of PpIX and TPZ were injected intratumorally into experimental animals once a week at the dosage of 100 μg and 50 μg, respectively, two times. (a) Schematic of experimental design to examine the effectiveness of nanoVectors on tumor shrinkage. (b) Tumors of experimental groups removed from the scarified mice at the study end point, Day 14 (14 days after treatment). (c) Quantitative evaluation of tumor growth for 14 d after treatment with PBS or various formats of PpIX and/or TPZ. TPZ@LXL-1-PpIX-MMT-2 treatment delayed tumor growth (*$P<0.05$). (d) Quantitative evaluation of animal body weight for 14 d after treatment with PBS or various formats of PpIX and/or TPZ. (e) H&E staining and immunohistochemical analysis for tumor hypoxia. The changes in cell density (upper panel) and cellular morphology in hypoxia (bottom panel) were apparent (as indicated in arrows). No drug other than PBS was received by control group.
